# Cholesterol Biosynthesis Inhibitor RO 48–8071 Suppresses Growth of Epithelial Ovarian Cancer Cells in Vitro and In Vivo

**DOI:** 10.26502/jcsct.5079185

**Published:** 2023-01-09

**Authors:** Yayun Liang, Kenneth P Nephew, Salman M Hyder

**Affiliations:** 1 Dalton Cardiovascular Research Center, University of Missouri, Columbia 65211, United States; 2 Dept of Biomedical Sciences, University of Missouri, Columbia 65211, United States; 3 Indiana University School of Medicine, Bloomington, IN 47405, United States

**Keywords:** Ovarian cancer, Cholesterol biosynthesis, Growth inhibition, Xenografts

## Abstract

**Introduction:**

Epithelial Ovarian Cancer (EOC) cells express enzymes in the cholesterol biosynthetic pathway, making this pathway an attractive therapeutic target for controlling ovarian cancer. Potent small molecule inhibitors of one biosynthetic enzyme, Oxidosqualene Cyclase (OSC), have been identified, and RO 48–8071 (4′-[6-(allylmethylamino)hexyloxy]-4-bromo-2′-fluorobenzophenone fumarate) (RO), has emerged as a useful chemotherapeutic agent for breast and prostate cancer.

**Methods:**

Cell viability assays were performed to determine effects of RO 48–8071 on growth of EOC cells. Aldehyde Dehydrogenase (ALDH) assay was conducted to determine the effects of drug on reducing stem cell like properties of EOC cells. Finally, xenograft studies were performed to assess the ability of RO 48–8071 to inhibit the growth of EOC cells in vivo.

**Results:**

We found that short-term (24–48 h) administration of pharmacological doses of RO effectively reduced the viability of drug-resistant EOC cells (SK-OV-3 and OVCAR-3), as determined with sulforhodamine B colorimetric assays. In 7-day assays, nanomolar concentrations of RO effectively inhibited the growth of EOC cells. RO also suppressed ALDH activity, a marker of stem cells. Importantly, RO significantly suppressed growth of xenografts derived from EOC cells when given to mice intraperitoneally (20–40 mg kg^−1^ day^−1^) for 27 days once tumors reached 100 mm^3^ (controls: 336 + 60 mm^3^; treated: 171 + 20 mm^3^) with no toxicity to the experimental animals. Mechanistically, RO induced apoptosis in tumor cells in vivo as shown with immunohistochemistry.

**Conclusion:**

Cholesterol biosynthesis inhibitor RO 48–8071 is thus a novel and potent inhibitor of human EOC, including EOC stem cells.

## 1. Introduction

Epithelial ovarian cancer (EOC) is an aggressive and deadly disease and despite concerted efforts to develop new strategies for preventing and treating the disease, almost 25,000 new cases are reported each year in the United States [1]. Those afflicted with EOC have a poor prognosis due to drug resistance and metastasis, resulting in the highest mortality rate of all known gynecological malignancies [2–4]. EOC is often detected at an advanced stage, associated with multifocal intraperitoneal dissemination, and accompanied by intense neovascularization [3,4]. Patients usually die due to drug-resistant, meta-static disease [3–5]. Consequently, both treatment and prevention strategies for ovarian tumors are needed.

Cholesterol is an essential structural and functional component of cellular membranes and cellular metabolism and is vital for tumor growth [6]. Tumor cells reprogram cholesterol metabolism to their advantage for growth, and thus provide several targets that could be exploited to control cholesterol production and tumor growth [7,8]. Targeting cholesterol biosynthesis is an active area of cancer therapeutics [7–9]. Statins are used to inhibit HMG-CoA reductase, an enzyme that is essential for cholesterol biosynthesis, but statin therapy causes a number of undesirable side effects that can be attributed to reduced levels of isoprenoids, defective post-translational modification of membrane proteins, and impaired membrane structure and function. Thus, alternative approaches to inhibiting cholesterol biosynthesis are being considered. 2, 3-Oxidosqualene Cyclase (OSC), which converts 2, 3-monoepoxysqualene to lanosterol, a key step in the biosynthesis of cholesterol, has recently emerged as a possible new target for inhibiting the cholesterol biosynthetic pathway [10] and potent small molecule inhibitors of OSC have been identified. Because OSC functions downstream of HMG-CoA reductase during cholesterol biosynthesis, it is unlikely that its inhibition will cause the adverse effects associated with statins. Further-more, cholesterol is both a metabolic precursor for endogenous steroid hormones, including estradiol, and itself can be modified to 27-hydroxycholesterol to increase estrogen signaling in some tissues, including in a subset of EOC cells [11] and contribute to disease progression.

EOC cells express enzymes involved in the biosynthesis of endogenous cholesterol [12,13]. We hypothesized that inhibition of cholesterol synthesis may be an effective means by which to arrest EOC cell proliferation. In this study, we tested a potent OSC inhibitor, RO 48–8071 (RO) [10,14] to determine its effectiveness against EOC cell lines, con-ducted studies to ascertain the ability of RO to inhibit EOC stem-cell-like properties and investigated the in vivo anti-tumor effects of RO in mouse xenograft studies. We propose that RO alone or in combination with chemotherapeutic drugs may provide a novel and hitherto untested approach to combating the progression of human EOC.

## 2. Materials and Methods

### 2.1 Cell lines and cell culture

Epithelial ovarian cancer cell lines OVCAR-3 (high grade serous) and SK-OV-3 (non-high grade serous) were obtained from ATCC (Manassas, VA, USA). Human immortalized ovarian surface cells (IOSE) were acquired from Dr. K. Nephew, Indiana School of Medicine at Bloomington, IN, following previously described approaches [15]. All cells were grown in 100 mm tissue culture dishes and harvested with 0.05% trypsin-EDTA (Invitrogen Corporation & Life Technologies, Grand Island, NY, USA). Cell-specific media were obtained from invitrogen and noted in the Figure legends 1–6.

### 2.2 Cell viability assay by SRB

Sulforhodamine B (SRB) assays were used to measure cell viability, as previously de-scribed [16,17]. Briefly, cells were grown to 70% confluence and, harvested with 0.05% Trypsin-EDTA then seeded in 96-well plates in 100 μL 5% FBS (JRH Biosciences, Lenexa, KS, USA) growth medium and incubated overnight at 37°C with 5% CO_2_ (see specific details in the Figure legends 1–6). Cells were washed once with medium without FBS and incu-bated for 24 or 48 h in 100 μL growth medium containing 5% FBS in the presence of different concentrations of RO (Tocris BioScience, Bristol, United Kingdom; Catalogue number: 5389; soluble in water up to 20 mM). Surviving or adherent cells were fixed in situ by adding 100 μL 50% cold trichloroacetic acid for 1 h at 4°C. Cells were washed with ice water, dried, and stained with 50 μL 4% SRB (Sigma, St. Louis, MO, USA) for 8 min at room temperature. Unbound stain was removed by washing cells 5× with cold 1% acetic acid, after which the cells were dried at room temperature. Cell-bound stain was solubilized in 150 μL 10 mM Tris buffer and quantified at 520 nm using a SpecTRA MAX 190 microplate reader (Sunnyvale, CA, USA). Six wells were used for each dose/experimental condition, and each experiment was performed at least twice.

### 2.3 FACS analysis for ALDH activity

EOC cells were washed once with Phosphate-Buffered Saline (PBS; Invitrogen) and harvested using Accutase (BD Biosciences, Franklin Lakes, NJ, USA). Aldehyde Dehydrogenase (ALDH) activity was measured using an Aldefluor^™^ kit (STEMCELL Technologies, Vancouver, BC, Canada) as described previously [18,19], and Fluorescence-Activated Cell Sorting (FACS) was performed per the manufacturer’s protocol. The ALDH inhibitor N,N-Diethylaminobenzaldehyde (DEAB) was added to the cultures as a negative control.

### 2.4 Animal xenograft tumor studies in nude mice

Female athymic nu/nu nude mice, 5–6 weeks old (18–22 g) were purchased from Envigo Inc. (Indianapolis, IN, USA). The mice were housed in a laminar air-flow cabinet under specific-pathogen-free conditions. All facilities were approved by the American Association for Accreditation of Laboratory Animal Care in accordance with the current regulations and standards of the United States Department of Agriculture, the Department of Health and Human Services, and the NIH. All procedures were approved by the institutional animal care and use committee.

Cells were harvested by trypsinization, washed twice with DMEM/F12, and re-suspended in 150 μL DMEM/F12 medium mixed with Matrigel (50% v/v; BD Biosciences, Bedford, MA, USA). EOC cells (SK-OV-3; 6 × 10^6^) were injected subcutaneously into each flank of experimental mice (n = 7–8). Tumors were measured every 3 days with a digital caliper, and tumor volumes were calculated using the following formula: (L × W × H) × π/6) [20].

### 2.5 RO treatment of xenografts

Animals were randomly assigned to three groups of 7–8 mice each. Once the tumors reached approximately 150 mm^3^, animals were treated with RO (20 or 40 mg kg^−1^ day^−1^ ip), and the third group was injected with PBS, as a vehicle control as described previously [20]. A total of 27 treatments were given during treatment. Animals were weighed throughout the experiment, and no apparent toxicity was observed based on changes in body weight. Experiments were terminated by sacrificing the animals after the last injection.

### 2.6 TUNEL assay

The TUNEL assay and quantification was performed as described previously [20]. Briefly, an apoptotic index was calculated from tumor sections by analyzing six sections each from approximately 4–5 tumors/group (for a total of 24–30 sections/group) and approximately 300–500 cells from each section were analyzed. Tumors were taken from different animals. Necrotic areas were avoided to reduce errors in the results.

### 2.7 Statistical analysis

Statistical significance was measured by using Student’s *t*-test or one-way analysis of variance (ANOVA) with repeated measures over time. When necessary, it was assumed that ANOVA was non-parametric. Values are reported as mean ± Standard Error of the Mean (SEM). For samples with a significant F-ratio (p < 0.05), the Student-Newman-Keuls multirange test was employed (SigmaStat).

## 3. Results

### 3.1 RO suppresses growth of epithelial ovarian cancer cells at pharmacological and nanomolar levels *in vitro*

**Figure 1:**
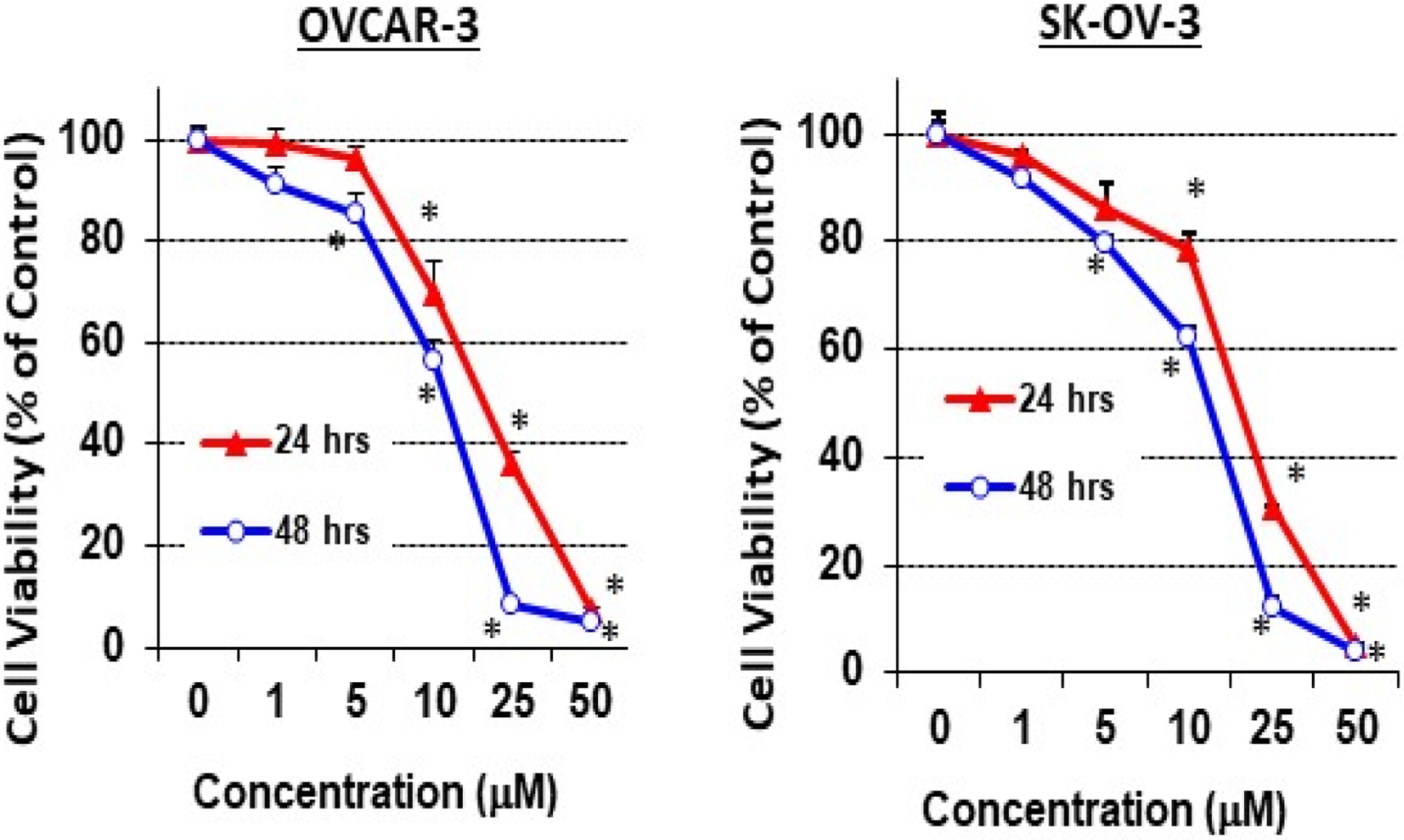
RO inhibits ovarian cancer cell growth *in vitro*. Ovarian cancer cells (OVCAR-3 or SK-OV-3, 6 × 10^3^/well) were seeded in 96-well plates (6 wells per concentration used) and incubated with different concentrations of RO 48–8071 for 24 or 48 h in RPMI-1640 medium + 10% FBS (OVCAR-3) or in McCoy’s 5a medium + 5% FBS (SK-OV-3). Control cells (0 μM) were incubated with vehicle (same medium + PBS). Cell viability was determined with an SRB assay. Values represent means ± SEM (n = 6). * Significant difference compared with the control group (set at 100%) (p < 0.05 using ANOVA).

**Figure 2:**
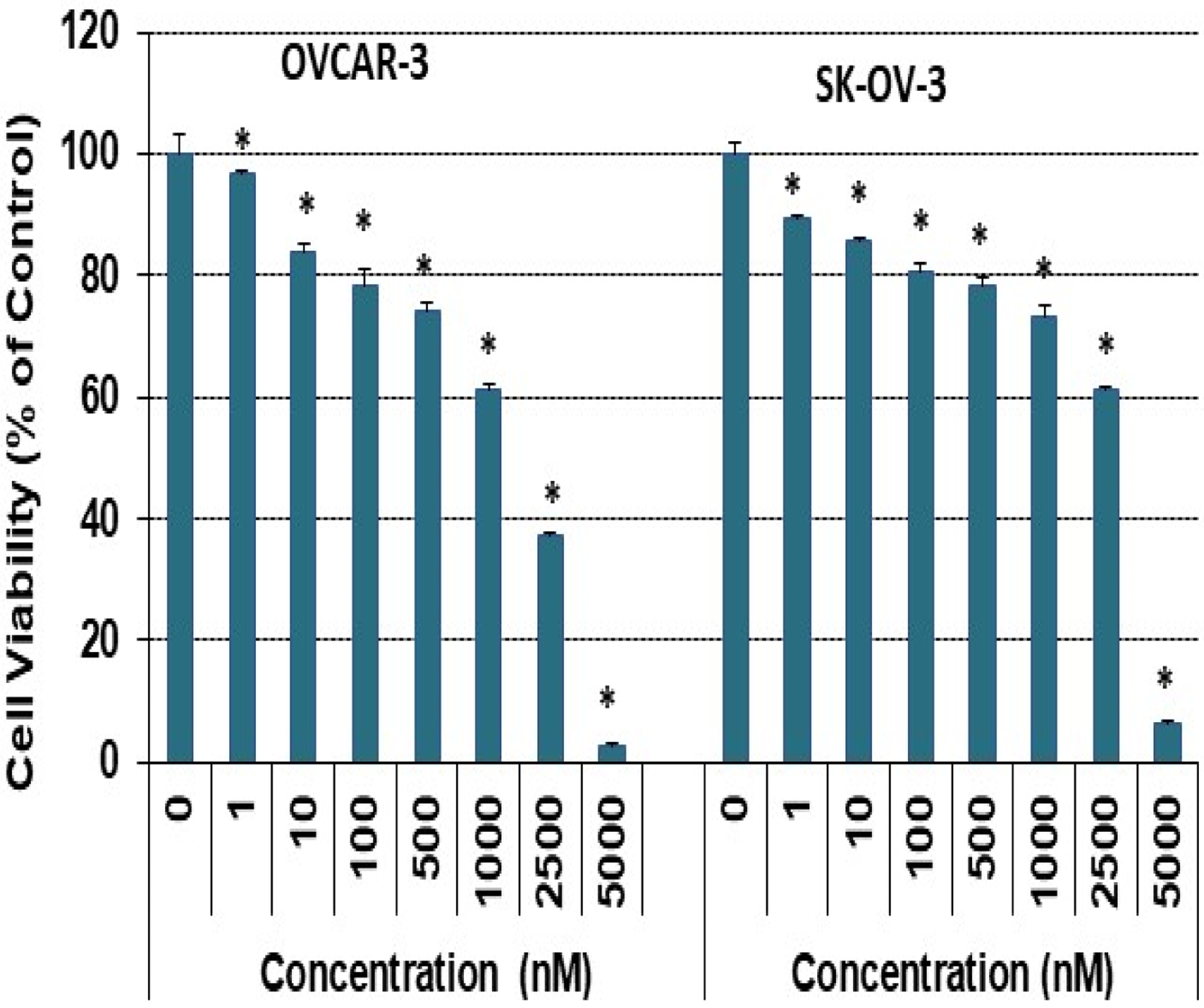
Low-dose RO inhibits ovarian cancer cell growth in vitro. OVCAR-3 ovarian cancer cells (8 × 10^**4**^/well) were seeded into 6-well plates in 10% FBS RPMI-1640 medium. SK-OV-3 cells (5.5 × 10^**4**^/well) were seeded into 6-well plates in 5% FBS McCoy’s 5a medium. Cells were treated with the indicated concentration of RO 48–8071 for 7 days. Cells were then re-treated with the same concentration of RO 48–8071 every other day. Control cells were incubated with vehicle (same medium + PBS). Cell viability was determined by SRB assay as described in the Methods. Three or four wells were used for each concentration, and each experiment was performed twice. * Significant difference compared with the control group (0 nM). (p < 0.05 using ANOVA).

A pharmacological dose of RO reduced the viability of both OVCAR-3 and SK-OV-3 EOC cell lines (Figure 1A). The IC_50_ values for inhibition of cell viability were 20.5 ± 0.3 μM and 18.3 ± 0.6 μM for OVCAR-3 and SK-OV-3 cells respectively, in a 24-h SRB assay; IC_50_ values were reduced to 11.3 ± 0.3 μM and 12.7 ± 0.5 μM in a 48-h SRB assay (Table 1). We also determined whether lower concentrations of RO given over a longer period might be effective. Concentrations as low as 1 nM effectively reduced cell viability in a 7-day SRB assay (Figure 2), indicating that clinically useful levels of RO could be achieved over an extended time frame to reduce the growth of ovarian cancer cells that are generally resistant to some commonly used chemotherapeutic drugs.

### 3.2 RO does not arrest growth of normal ovarian cells

**Figure 3:**
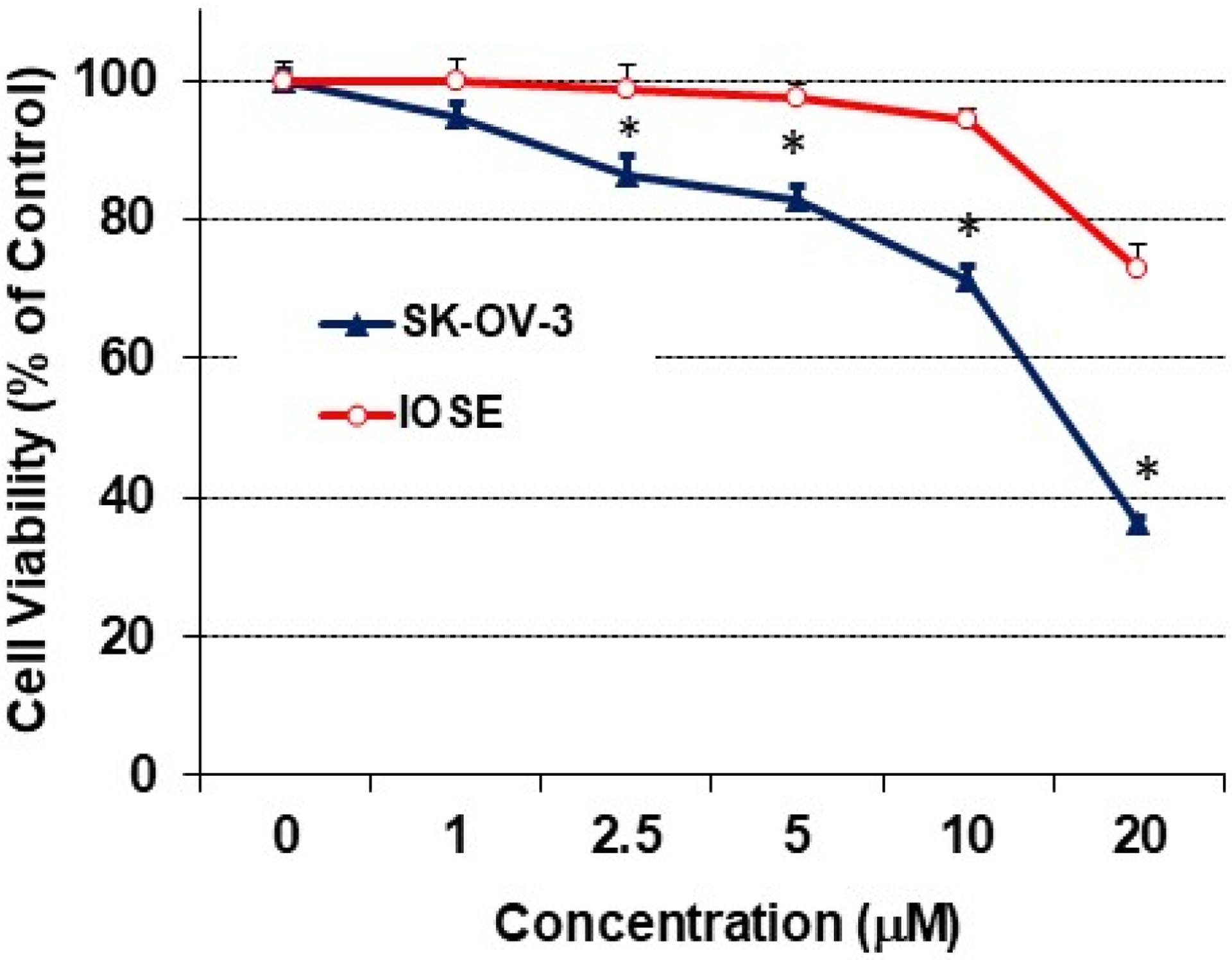
The effect of RO on growth of normal ovarian cells. Normal ovarian IOSE cells (5 × 10^3^/well) were seeded into 96-well plates and grown in NOSE medium (medium 199 and medium 105, 1:1, supplemented with 10% FBS, 10 μg/mL EGF, 200 μg/mL hydrocortisone, and 100 × penicillin-streptomycin-glutamine) overnight. The next day, the cells were washed and treated with the indicated concentration of RO 48–8071 in 5% NOSE medium for 24 hours. Control cells (concentration of 0 μM) were incubated with vehicle (same medium + PBS). Cell viability was determined with an SRB assay and each bar represents values derived from 6 wells for each concentration and controls; values represent means ± SEM (n = 6). Experiment was performed twice. *Significant difference between IOSE and SKOV-3 cells at each concentration (p < 0.05 using *t*-test).

In order to determine whether RO might be toxic to non-cancerous cells, we exposed normal IOSE ovarian cells to RO and compared its effects with those on SK-OV-3 ovarian cancer cells. Concentrations of RO up to 10 μM had no effect on the viability of normal IOSE cells, as determined by ANOVA (Figure 3). However, RO did affect tumor cells, 30% of which were not viable in the presence of 10 μM RO.

### 3.3 RO reduces stem-cell-like properties of ovarian cancer cells

**Figure 4:**
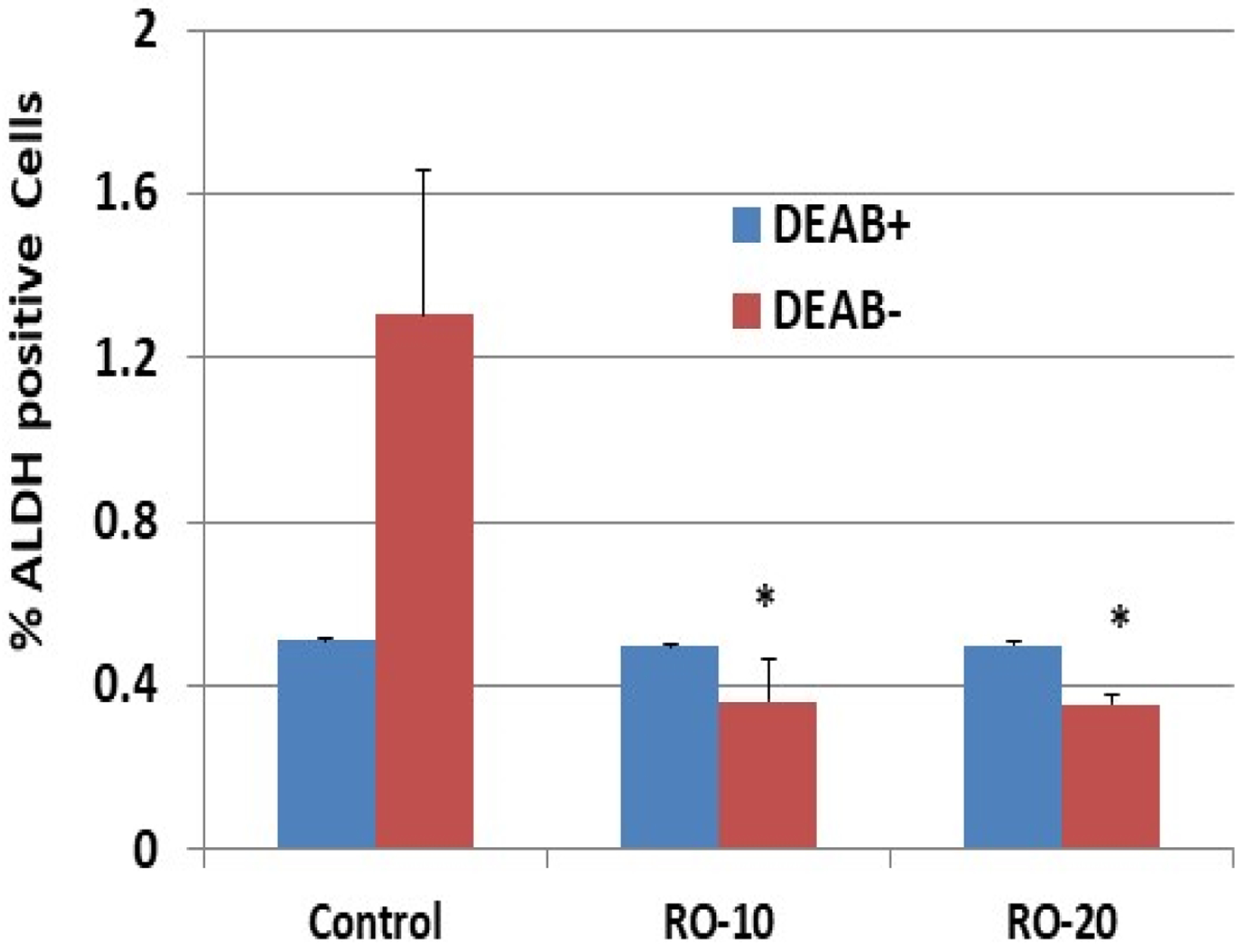
RO inhibits ALDH expression in SK-OV-3 ovarian cancer cells. An Aldefluor kit was used for identification of cancer stem cells, which express high levels of ALDH. SK-OV-3 cells were grown in 100-mm culture dishes and treated with the indicated concentrations of RO 48–8071 (10 μM or 20 μM in 5% FBS McCoy’s 5a medium) for 24 hours (n=3 dishes per RO concentration). Cells (2.5 × 10^4^/sample) were mixed with BAAA (from the Aldefluor kit) with or without DEAB (an inhibitor of ALDH) and incubated for 40 minutes before being suspended in the Aldefluor assay buffer. Fluorescence was measured using a FACSCalibur and analyzed with Summit V5.2.0.7477 software. The ALDH^+^ subpopulation was obtained in the absence of DEAB. The values represent % of ALDH-positive cells ± SEM. *Significant difference compared with the control DEAB– group (p < 0.05 using ANOVA).

Many ovarian cancer cells display stem-cell-like properties, including high levels of the enzyme ALDH [21]. We used FACS to determine whether RO inhibits ALDH activity in SK-OV-3 cells. Cells treated overnight with 10 or 20 μM RO had substantially less ALDH than cells treated with PBS alone (controls) (Figure 4). As expected, cells treated with DEAB showed lower levels of ALDH positivity in the controls and no change when exposed to either concentration of RO.

### 3.4 RO suppresses growth of human epithelial ovarian cancer xenografts *in vivo*

**Figure 5:**
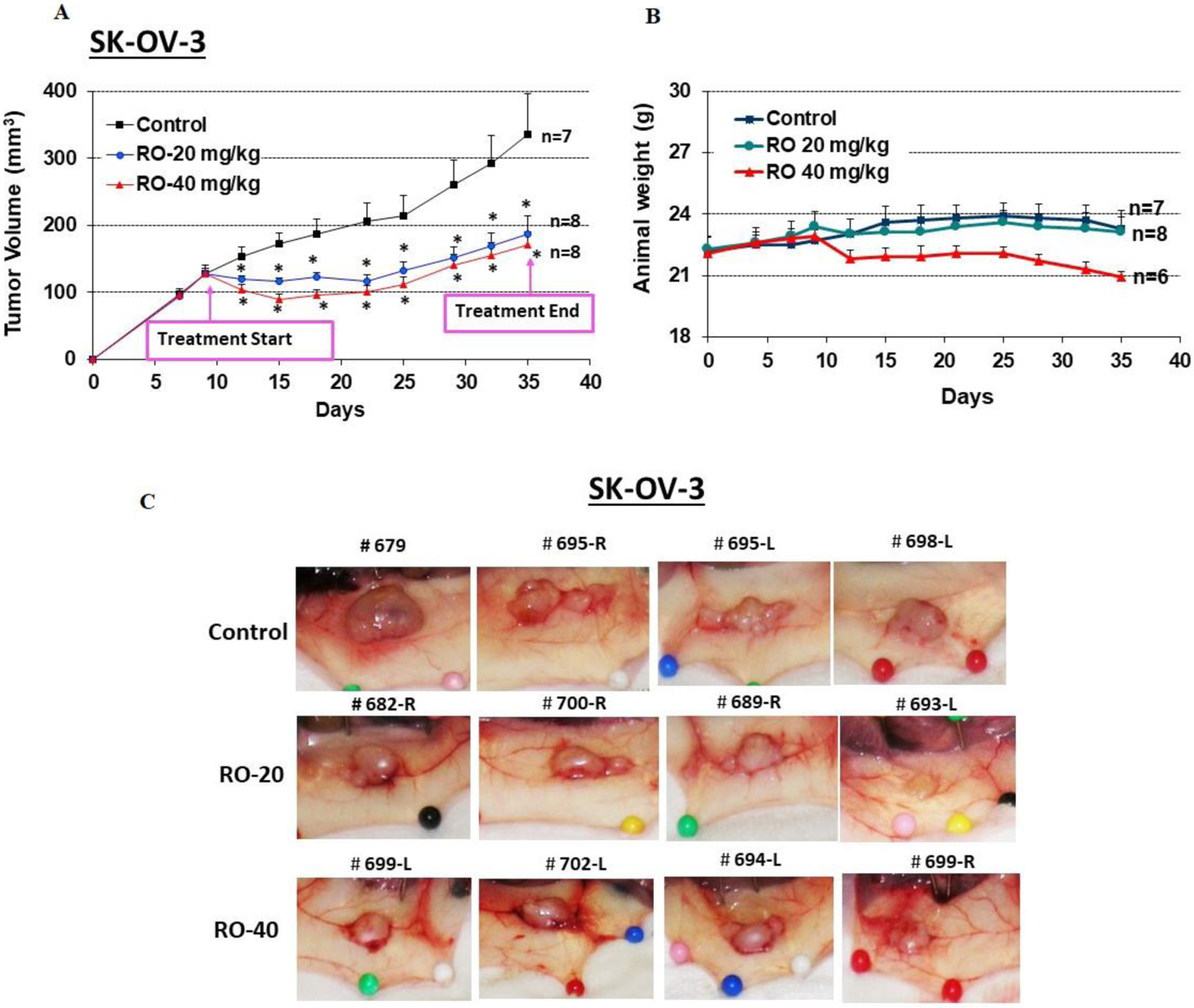
RO inhibits SK-OV-3 tumor xenograft growth *in vivo*. SK-OV-3 ovarian cancer cells (6 × 10^6^) in 0.15 mL of Matrigel:McCoy’s 5a medium (4:1 v/v) were injected into each flank of nude mice (two sites per mouse). When tumor volumes reached approximately 150 mm^3^, animals were assigned to one of three groups and injected ip daily with vehicle (PBS), RO 20 mg/kg, or RO 40 mg/kg for 27 days. (A) Tumor volumes were monitored throughout the experiment. Values represent mean ± SEM (n = 7–8 animals/group). * Significant difference compared with the control group (p < 0.05 using ANOVA). (B) Animal weight was monitored throughout the experiment. Values represent means ± SEM (n = 7–8 animals). There were no significant differences among control and treatment groups in B. (C) Representative tumors shown in each group.

We next determined whether RO suppresses the growth of EOC cells *in vivo*. SK-OV-3 cells were injected subcutaneously into both flanks of nude mice, which were subsequently treated daily with PBS (control) or 20 or 40 mg/kg RO ip for 27 days. RO significantly suppressed the growth of the tumor xenografts derived from SK-OV-3 ovarian cancer cells (171 ± 20 mm^3^ versus 336 ± 60 mm^3^ for the control after 27 days; p < 0.05, ANOVA; Figure 5A). The animals showed no significant weight loss during the treatment protocol (Figure 5B). Examples of tumors in control and treatment groups are shown in Figure 5C. RO similarly suppressed growth of OVCAR-3 cells in vivo (data not shown).

### 3.5 RO induces apoptosis in epithelial ovarian cancer cells *in vivo*

**Figure 6:**
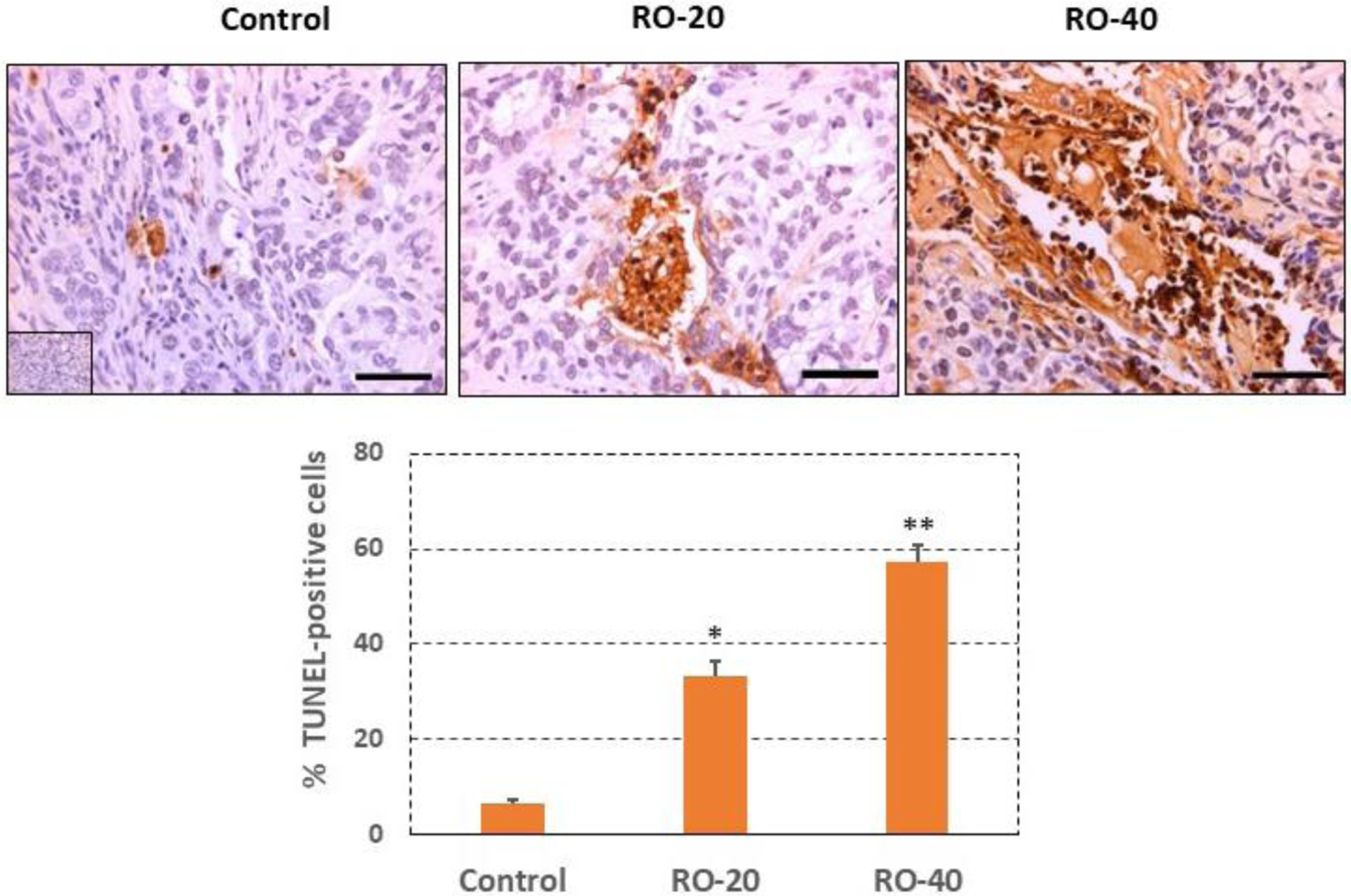
RO induces apoptosis in SK-OV-3 tumor cells and cell death *in vivo*. Tumors were collected at the end of the experiment shown in Figure 5 and processed for immunohistochemistry to measure the TUNEL signal as described in the Methods. The upper panel shows the TUNEL signal in representative sections taken from tumors. The mean % TUNEL-positive cells per group are shown in the lower panel (bar graph). Values represent mean ± SEM of TUNEL-positive cells in 24–30 tumor sections/group in tumors from 4–5 individual tumors taken from different animals. Approximately 300–500 cells were analyzed per section. Inset represents negative staining control. Bars in the sections represent 50 μm. *Significant difference compared with the control group, p < 0.05; ** Significant difference compared with the Control and RO-20 groups, p< 0.05.

To investigate the mechanism of tumor suppression, we performed TUNEL assays in tumor sections to assess apoptosis. RO induced apoptosis in EOC cells *in vivo* (Figure 6) which likely reduced tumor volumes. Several areas of extensive necrosis in the tumors treated with 40 mg/kg RO were not included in the analysis to avoid errors in counting apoptotic cells. While 40 mg/kg treatment led to the detection of more apoptotic cells in tumors compared with 20 mg/kg treatment, this was not reflected in any differences in tumor volume. It is our contention that this could be due to the time of tissue collection in this experiment, which did not allow for tumor volumes to shrink even though treatment with 40 mg/kg RO initiated far greater levels of apoptosis compared with the lower treatment. Little tumor volume change was seen in Figure 5C, which represents tumors *in situ*.

## 4. Discussion

EOC is an extremely aggressive form of cancer which often has a poor prognosis due to drug resistance and metastasis [2,3,22]. It is now well established that cholesterol bio-synthesis plays a major role in the growth of many different types of tumors, providing various biosynthetic enzymes that could be targeted pharmaceutically to suppress the production of cholesterol and hence reduce tumor growth [7–9]. The enzyme 2,3-Oxidosqualene Cyclase (OSC) has recently emerged as a possible new target for inhibiting this pathway [7/10], as it performs a key step in the cholesterol biosynthetic pathway, by converting 2, 3-monoepoxysqualene to lanosterol. While many studies have concentrated on the role of statins, which control HMG CoA reductase, the rate limiting enzyme in cholesterol biosynthesis, we examined whether targeting OSC might be even more beneficial.

In the studies reported herein, we observed that an inhibitor of OSC, RO, potently suppressed the growth of EOC cells, both in vitro and in vivo. Initially we studied the effects of RO in vitro on two aggressive EOC cell lines, high grade serous OVCAR-3 and non-high grade serous SK-OV-3, and established that the inhibitor reduced the viability of both cell lines. Although the SRB procedure used was designed to determine cell viability [16,17], it is our contention that RO was killing the cells directly, as also noted later in the in vivo studies. The rapid suppression of tumor cells in response to treatment with RO supports the notion that RO does indeed destroy tumor cells, though this needs to be confirmed in vitro. Inhibition occurred when cells were exposed to pharmacological levels of the drug, and also when a more therapeutically relevant dose in the nanomolar range was used in long-term assays.

Based on our observations, we could foresee the potential clinical infusion of low levels of RO to OC patients, though further preclinical testing is clearly necessary to ensure there are no toxic effects of the drug. It is encouraging to note that RO at concentrations up to 10 nM did not affect normal ovarian cells that were tested in the same cell viability assay, suggesting that it may be non-toxic when administered clinically at low doses in the nM range, which were also found to be effective over a longer time in vitro. Animals treated with RO did not exhibit changes in weight, further supporting the view that RO has no significant toxic side-effects in vivo. These results are encouraging, and we are optimistic that the anti-tumor properties of RO might be harnessed to treat EOC.

Because EOC cells are enriched with stem cells [23,24] we determined whether RO might also suppress stem cell activity. The cholesterol biosynthesis inhibitor potently suppressed ALDH activity, a marker of stem cells, in SK-OV-3 cells [25]. This suggests that treatment of existing tumors with RO could reduce the number of stem cells and thereby prevent tumor re-emergence, though this needs further confirmatory studies involving several tumor cell-lines, both in vitro and in vivo. The mechanism by which RO affects stem-cell markers is unclear and requires further study.

We extended our studies to the in vivo situation by examining the ability of RO to suppress subcutaneous tumors derived from SK-OV-3 EOC cells in a nude mouse xenograft model. Following injection of SK-OV-3 cells into both flanks, animals were given either 20 or 40 mg/kg RO for 27 days. Both doses effectively suppressed tumor growth, and tumor size remained static throughout the treatment timeframe due to tumor cells under-going apoptosis. While 40 mg/kg RO induced greater levels of apoptosis, tumor volumes in the two treatment groups remained the same, indicating that perhaps there was insufficient time for tumors to shrink in the 40 mg/kg dose group. It also appeared that following the initial response to RO, tumors became resistant and began to grow again. This suggests that combination therapy using RO in tandem with additional chemotherapeutic agents might be necessary to control progression of ovarian cancer cells in vivo.

Mechanistically, our data showed that RO induced apoptosis of ovarian tumor cells in vivo, as it does with breast and prostate tumor cells [26–28] although its mechanism of action as an anti-tumor agent, as well as its ability to suppress cholesterol biosynthesis in ovarian cancer cells requires further investigation. It also remains to be determined whether RO can potentially function as a preventive agent by reducing cholesterol synthesis in the early phases of tumor development, if the disease is detected earlier, which could result in the suppression of tumor growth beyond a very small volume.

## 5. Conclusions

In conclusion, our data show that the cholesterol biosynthesis inhibitor RO effectively suppresses the growth of both high grade and non-high grade EOC cells, including EOC with stem-cell like properties, and induces apoptosis in vivo. As reported previously [29] RO may also function as an anti-angiogenic agent, while also degrading steroid receptors that could be involved in EOC cell growth [26,27]. However, it appears that following treatment, some tumors begin to develop resistance to RO, as shown by increasing tumor volumes in the later stages of treatment, suggesting that combination therapy using RO together with existing or new therapeutic compounds might more effectively control this deadly disease.

## Figures and Tables

**Table 1:** IC_50_ values for inhibition of cell viability.

Drug	Cell lines	IC_50_ (μM) (24 hours)	IC_50_ (μM) (48 hours)
Ro 48–8071	OV-CAR-3	20.51 ± 0.33	11.29 ± 0.33
	SK-OV-3	18.28 ± 0.64	12.72 ± 0.52

## Data Availability

All datasets presented in this study are included in the article. Any other information can be obtained from authors on request.

## Abbreviations:

ALDH: Aldehyde Dehydrogenase
ANOVA: Analysis of Variance
DEAB: N,N-Diethylaminobenzaldehyde
EOC: Epithelial Ovarian Cancer
FACS: Fluorescence-Activated Cell Sorting
IOSE: Immortalized Ovarian Surface Cells
OSC: Oxidosqualene Cyclase
PBS: Phosphate-Buffered Saline
RO: RO 0488071 (4′-[6-(allylmethylamino)hexyloxy]-4-bromo-2′-fluorobenzophenone fumarate)
SEM: Standard Error of the Mean
SRB: Sulforhodamine B
TUNEL: Terminal Deoxynucleotidyl Transferase Dutp Nick End Labeling.
